# The “Good Cop, Bad Cop” Effect in the RT-Based Concealed Information Test: Exploring the Effect of Emotional Expressions Displayed by a Virtual Investigator

**DOI:** 10.1371/journal.pone.0116087

**Published:** 2015-02-20

**Authors:** Mihai Varga, George Visu-Petra, Mircea Miclea, Laura Visu-Petra

**Affiliations:** 1 Applied Cognitive Psychology Center, Department of Psychology, Babeş-Bolyai University, Cluj-Napoca, Romania; 2 Developmental Psychology Lab, Babeş-Bolyai University, Cluj-Napoca, Romania; 3 COGNITROM Ltd, Cluj-Napoca, Romania; University of Udine, ITALY

## Abstract

Concealing the possession of relevant information represents a complex cognitive process, shaped by contextual demands and individual differences in cognitive and socio-emotional functioning. The Reaction Time-based Concealed Information Test (RT-CIT) is used to detect concealed knowledge based on the difference in RTs between denying recognition of critical (*probes*) and newly encountered (*irrelevant*) information. Several research questions were addressed in this scenario implemented after a mock crime. First, we were interested whether the introduction of a social stimulus (facial identity) simulating a virtual investigator would facilitate the process of deception detection. Next, we explored whether his emotional displays (friendly, hostile or neutral) would have a differential impact on speed of responses to probe versus irrelevant items. We also compared the impact of introducing similar stimuli in a working memory (WM) updating context without requirements to conceal information. Finally, we explored the association between deceptive behavior and individual differences in WM updating proficiency or in internalizing problems (state / trait anxiety and depression). Results indicated that the mere presence of a neutral virtual investigator slowed down participants' responses, but not the appended lie-specific time (difference between probes and irrelevants). Emotional expression was shown to differentially affect speed of responses to critical items, with positive displays from the virtual examiner enhancing lie-specific time, compared to negative facial expressions, which had an opposite impact. This valence-specific effect was not visible in the WM updating context. Higher levels of trait / state anxiety were related to faster responses to probes in the negative condition (hostile facial expression) of the RT-CIT. These preliminary findings further emphasize the need to take into account motivational and emotional factors when considering the transfer of deception detection techniques from the laboratory to real-life settings.

## Introduction

The quest for identifying objective indicators of deceptive behavior represents a flourishing field of scientific inquiry. Although behavioral cues represent an obvious candidate for deception detection, the traditional approach has largely overlooked the cognitive processes involved in producing and executing secretive behaviors. More recently, it has been convincingly argued that focusing on cognitive processes would be an essential step in the development of a mechanistic understanding of deception [1], and in improving the existing deception detection techniques [2, 3].

A growing body of literature investigating the cognitive underpinnings of deceptive behavior suggests that it requires greater cognitive effort compared to truthful responses [4], [2], [5], with the truth being considered an automatic, default-like state of the cognitive system [6]. When deceiving, one has to coordinate several cognitive demanding tasks: to select a response that is incompatible with the truth, to suppress a recurring awareness of the truthful information, to compile alternatives, while maintaining the consistency of the lie, to monitor personal behavior and the reaction of the audience to the deception [7]. These actions rely on many cognitive mechanisms that must be activated and integrated successfully, such as: planning, inhibition, working memory (WM), and cognitive flexibility [8, 9, 10, 11]. In the literature, these mechanisms have been generally subsumed under the construct of executive functions. Given that deception is a complex cognitive process, such different mechanisms are likely to interact with each other, supporting the seminal “independent and interdependent” view of executive functioning proposed by Miyake and collaborators [12, 13]. However, the theoretical positions which convincingly argue for the involvement of executive functions in deceptive behavior need to be empirically documented, especially in the light of recent literature suggesting that when it is practiced, deceptive behavior is not necessarily more effortful than truthful one [14], and that it is amenable to training programs which can substantially increase its efficiency [15, 16].

So far, three types of approaches have been used to (indirectly) demonstrate the involvement of executive functions in deceptive behavior: *neurocognitive studies* which reveal the activation of a similar network to that involved in solving executive-demanding tasks [9, 17], *individual differences* approaches which link personal strengths and weaknesses in executive functioning to deception efficiency [18–20], and *interference designs*, which selectively disrupt executive functioning by introducing a concurrent executive task, and assess the impact of this manipulation on deceptive behavior [21–25].

Based on the findings of such studies, we decided to further explore the relationship between deception and *WM updating*, since all the approaches mentioned above pointed to its specific involvement in deceptive behavior. More specific, the meta-analysis by Christ et al. [9] showed a significant overlap between brain regions involved in deception and those specifically involved in WM (but not in inhibition or task switching), suggesting that WM could play a “particularly important role in deception” (p. 1563). However, individual differences approaches have revealed a negative relationship between individual strengths in verbal/spatial WM and deception efficiency [19, 26], probably as a result of the truthful content being more easily accessed and reinforced in individuals with better WM skills. Interference designs have yielded mixed findings, revealing that the introduction of a concurrent WM task in parallel with the deception task affected RTs to critical items to a larger extent compared to responses to irrelevant items [22], but revealing a less discriminatory power of this manipulation compared to the introduction of a concurrent set switching demand [24].

Given the variability of the specific methodologies used to generate deceptive behavior throughout the abovementioned studies, it is not surprising that few consistent findings have emerged. The use of a unitary methodology could lead to a better understanding of the role played by WM updating in deceptive processes. An ideal candidate for such an approach is the Concealed Information Test (CIT, previously known as the Guilty Knowledge Test) introduced by Lykken [27, 28] as a psychophysiological test revealing “memory traces” [29] of the information that the subject is trying to conceal. Although integrated in the deception detection literature, the CIT does not aim to identify deception per se, but rather investigates the possession of information that the participant is not willing to disclose [30]. Recently, behavioral measures (reaction times) were introduced in this paradigm [31], resulting in the now known RT-CIT [32, 33]. In this test, the examinee is required to give fast responses to three types of items: probes, targets, and irrelevant items. *Probes* represent crime-related details that the suspect could not miss to identify or notice; *irrelevant* items are several times more numerous and they share a variable degree of categorical similarity with the relevant items; *target* items are used in order to prevent the subject from entering into an automatic mode of responding, being also categorically similar with the other two types of items. In the RT-based CIT, an innocent subject will not be able to distinguish crime-related items from irrelevant items. Only guilty/knowledgeable subjects will recognize the critical items, thus allowing the examiner to link the knowledgeable suspect with the crime. It is thus expected that guilty or knowledgeable participants would present longer RTs (an appended “lie-specific time”, [26]) in denying recognition of probes, compared to irrelevant items, this constituting the concealed information effect. Reduced accuracy for those critical responses has also been documented in the already numerous laboratory studies confirming the validity of RT-based CIT [19, 25, 31–35].

### Social and emotional influences on detecting concealed information

Most of the CIT research usually relies on testing subjects in an environment lacking of *social* stimuli, despite the fact that concealing information is mostly a social action that amplifies the emotional involvement and anticipated consequences for the deceptive agent. [36] firstly addressed this gap and provided arguments for introducing social stimuli into the CIT. In real life, most interrogations take place in an environment that is saturated with social stimuli. The mere presence of an investigator triggers various physiological and behavioral responses, not to mention the role played by the ongoing feedback provided by such an individual in response to deceptive responses.

Indeed, previous studies have shown that the presence of another person during an experimental task may increase arousal and motivation [37, 38], and may also serve as a source of cognitive distraction [39] or as a source of cognitive load [40]. Such variables have already been linked to CIT findings. Recent studies have shown that arousal [41], motivation [41–43] and cognitive load [22, 24, 25] may influence one’s performance in concealing information. As Ambach et al. [36] indicated, this influence could go in multiple directions. Firstly, the motivation to remain undetected may be enhanced by the presence of the investigator, but it could also facilitate the motivation to confess. Secondly, the presence of another person could help the participant maintain his focus on the task, or it could divert his attention away from it. Thirdly, the presence of the investigator may increase the emotional involvement of the participant by creating a more intense conflict between disclosure and concealing information, or it may lower the arousal of the participants as a result of withdrawal and alienation. Some of the social facilitation research [44] indicated that visual presence of another person might lead to increased performance of truthful responses, but to impaired delivery of deceitful responses. In support, Harrison et al. [45] found that under certain circumstances, deceitful answers take extra time to deliver in the presence of others. The visual presence increased the variability in response latencies, and deceptive answers became hesitant and lengthy [45].

In an exploratory study, it has been suggested that the introduction of a virtual investigator in the CIT would lead to an increased involvement and enhanced arousal [36]. Results confirmed that a uniform male face presentation and voice accompanying each CIT item enhanced differential physiological and behavioral responding to critical items compared to responses to irrelevants. The authors proposed that attentional demands and participants’ motivation to avoid detection might be the important links between social stimuli and physiological responses in the CIT [36]. While the CIT has been considered relatively invulnerable to the effects of the stress experienced by the examinee [29], other variables such as motivation to avoid detection have been shown to influence responses, leading to an increased CIT effect [43]. Moreover, a study by Elaad [46] showed that a simple cognitive approach to CIT might be reductionist, since although in possession of identical information regarding critical items, guilty versus (informed) innocent participants showed enhanced physiological reactivity to crime-related objects.

The motivation and the individual’s drive to conceal the information may be increased by the presence of an embodied humanlike agent. Evidence from human–computer interaction has shown that even the presence of a static image of an agent can improve user motivation and can also increase engagement [47]. By using a male face presentation as the “virtual investigator” [36], we further assessed the influence of presenting social stimuli in the RT-CIT. Ambach et al. [36] suggested that future studies should disentangle the influence of *emotional* content of the social situation. By integrating an investigator’s face with different emotional displays, we can provide further information about how emotion interferes with the behavioral responses in the CIT. It is generally believed that the possible interference is due to the initial consumption of resources by emotional items or, possibly, due to an increased difficulty in disengaging from emotional information [48, 49]. However, there is surprisingly little research regarding the influence that the specific emotion displayed by the examiner has on detecting concealed information. While there is some speculation in the literature that a friendly attitude of the investigator makes the subject more willing to divulge important or sensitive information [50], to our knowledge, there is no research to test this prediction directly, either in a realistic interview, or in the controlled CIT context. On the contrary, displaying a negative emotional expression such as anger has been deemed to suggest either indiscriminate hostility or even to convey a presumption of guilt, associated with the so-called “investigator bias” which usually accompanies the “heavy-handed” tactics used by law enforcement professionals [51]. Again, we found no study directly assessing the impact of a negative (or neutral) affective display from a real or potential examiner on the process of concealed information detection.

### Current study

The current study envisions secretive behavior as an embodied and embedded process, placing it at the interplay between individual differences in cognitive and socio-emotional factors (executive functions, trait-like dimensions of depression, trait anxiety, or temporarily experienced state anxiety) and contextual dimensions (presence/absence of a virtual investigator displaying various emotional expressions). Several research questions were addressed through this design. First, we were interested whether *the simple introduction of a social stimulus simulating a virtual investigator would facilitate the process of deception detection*. Although this question has been already addressed in the Ambach et al. [36] study for the CIT based on physiological measurements, we wanted to extend this investigation to the RT-based CIT, anticipating that the effects of the social stimulus on deception detection would be enhanced by the speeded pace at which this task unfolds. However, we aimed to create a more simplistic (and analogue to real investigations) display of the CIT stimuli, so we introduced the examiner’s face immediately preceding, rather than simultaneous with the CIT item. Although this manipulation might have reduced the direct interference between the two (investigator face and CIT stimulus), a pilot study using a simultaneous display showed that it was very difficult for participants to keep up with the speeded pace of the complex RT-CIT items when both stimuli were present on the same display.

Next, we wanted to explore whether *the introduction of facial emotional displays of the virtual investigator* (happy, angry or neutral) *would have a differential impact on the responses to crime-relevant versus irrelevant items*. Considering the lack of previous studies in the deception detection literature, no specific hypotheses could be advanced regarding the impact of these emotional displays on the subjects’ deceptive or truthful behavior. However, it has been shown that the brief presentation of facial emotional expressions in a block of stimuli (hostile or friendly) could induce a corresponding transient, negative or positive mood in participants [52], which, in turn, could affect their RT-CIT behavior. According to affective network models and theories of motivational orientation [53], it has been argued that negative mood facilitates an avoidance tendency, while positive mood is characterized by a general approach tendency, regardless of the valence of the reference object. There is a growing body of evidence pointing to the facilitative effect of *positive* (compared to negative or neutral) affect on cognitive control in general [54, 55], although high emotionally distracting stimuli have also been shown to impair cognitive performance in typical and clinical samples [56]. To explain such inconsistent findings, Harmon-Jones and Gable [57] suggested that positive affect can narrow or broaden attentional control depending on how high in approach motivation an individual might be in the context of the task. They found that in situations of low approach motivation (e.g., after viewing pleasant pictures of cats), attention is broadened, while in situations of high approach motivation (e.g., after viewing pictures of desserts), attention is actually narrowed [58]. The presence of distracting *negative* information has also been shown to affect an individual’s ability to perform a task requiring cognitive control. Dolcos and McCarthy [59] revealed a disruption of the activity in brain regions involved in working memory maintenance when presented with negative emotional distractors, suggesting a potential competition between emotional and cognitive processes [60].

A secondary aim was to investigate whether the mood-inducing effects of the emotional expressions would be circumscribed to or amplified in the motivationally relevant RT-CIT context, compared to the effect that such emotional expressions would have on a memory updating task in which emotion would not have any social or motivational significance for the participants. Therefore, in the current study, by introducing emotional expressions as distractors in the context of the RT-CIT and in a well-known working memory paradigm (the *n*-back task) we explored whether the potential interfering (facilitative or disruptive) effect of this affective display would be similar, or whether it would differ across these two tasks. Administering a similar EM-*n*-back task to the one used in the present study, Ladouceur et al. [61] found that typically developing children presented significantly longer RTs to *n*-back items presented on positive backgrounds. Although the emotional facial display is just as task-irrelevant in the RT-based CIT as it is in the *n*-back task, we anticipated that it would disrupt performance to a greater degree in the former, considering the potential motivational valence attached to it as an emotional expression of a virtual investigator.

Finally, we were interested in the *modulating effect played by individual differences* in executive functions and trait-like predispositions to experience symptoms of depression or anxiety, as well as in the effects of state anxiety during the mock crime procedure of the CIT. The objective measure of memory updating in contexts containing or lacking emotional information (the EM-*n*-back task), plus a self-report measure of executive (dys)functions were introduced in order to test whether proficiency in memory updating and self-perceived difficulties/failures in everyday tasks requiring cognitive control would be linked to RT-CIT deception efficiency.

Individual differences in internalizing symptoms (especially in trait or state anxiety) have been linked to the way subjects process emotional information and to how this affects their performance on cognitive tasks (e.g. the Attentional Control Theory [62]. Previous studies have revealed their correlation with speed and accuracy of responses to probes, compared to irrelevant items in the context of the CIT or RT-CIT [19, 63]. Besides the well-documented biases in the processing of negative/threatening and positive information by high trait-anxious individuals, we also wanted to investigate the potential influence of higher levels of depression, which have also been related to increased attention for negative stimuli and decreased attention for positive stimuli [64], especially when such stimuli are presented for longer than 500 ms [65, 66]. Also, individual differences in reactivity to stress, besides levels of state/trait anxiety, could be responsible for an individual’s differential responses to critical items extracted from a mock crime, or to their vulnerability towards the emotional expressions of the examiner. However, to our knowledge, no previous study has related self-reported individual differences in depression and stress reactivity to responses on the RT-CIT. By introducing these self-report measures, we can test whether such individual differences would play a role in the standard RT-based CIT [19], and we can explore the influence of such individual differences on the new Em-RT-CIT.

## Materials and Methods

### Participants

Participants (N = 47, 11 males and 36 females; mean age 22.8 ± 5.1 years) were recruited from psychology undergraduate courses and received credit for their participation. Written informed consent was obtained directly from participants. All participants executed the mock crime procedure, followed by the RT-based CIT (four conditions) and the *n*-back (four conditions). Throughout the procedure, participants had to fill out four questionnaires, which are described below. Due to a technical failure, data from one participant were discarded from the analysis. Participants were debriefed at the end of the study and were informed about the nature of the experiment. The design of the study and the written consent procedure were approved by the ethics committee of the Faculty of Psychology and Educational Sciences, Babeș-Bolyai University, and designed in accordance with guidelines from the National College of Psychologists and international guidelines from the Declaration of Helsinki (1964) for research involving human subjects.

### Procedure

Upon arrival, all participants read and signed a consent form indicating the participation was voluntary and that they could withdraw at any time. Then they received and read the mock crime instructions, memorized the critical items (i.e. probes) and executed the assignment. After executing the mock crime, they completed a filler task consisting in two questionnaires (measuring state anxiety and executive functions) and lasting for approximately 15 minutes. Subsequently, participants learned the target items. Afterwards they were assessed with the four conditions of the RT-based CIT (one classical and three EM-RT-based CIT). Participants then completed the composite measure of depression, anxiety and stress. Finally, participants were tested with the Emotional *n*-back task (EM *n*-back, one without a facial background and three with facial backgrounds) and they filled out the trait anxiety questionnaire (see description below).

Mock Crime

Participants received written instructions about the mock crime procedure, so that all the relevant details were specified in advance. Each participant had to access the personal office of a university research assistant responsible for an upcoming exam. Upon entering the office, he/she had to search for a *memory stick* on which to copy the exam questions. In order to log into the computer, a password had to be used and the participants looked it up in an agenda found on the professor’s desk. After they copied the exam questions on the memory stick, they put it into a *laptop bag*. Participants were instructed to steal another three objects from the desk: a *mobile phone, a wireless mouse* and the *agenda*. Subjects read the written instructions twice and were instructed to memorize the five critical items (i.e. the probes). To increase the realism and the pressure of the scenario, they were also instructed to be fast and avoid being caught. Moreover, the testing scenario took place in a formal office, during program hours. After completing the mock crime, participants returned to the original room and were asked to verbally describe the physical characteristics of each stolen item in order to ensure a better encoding.

Next, the items were taken out of sight and the participants received a filler task, which lasted between 12 and 15 minutes. During this time, two questionnaires had to be filled out: STAI-Y1 (State Anxiety Inventory, [67]) to assess the state anxiety levels related to the mock crime scenario and the BRIEF (Behavior Rating Inventory of Executive Function, [68], see description below).

Reaction Time-based Concealed Information Test

**Fig 1:**
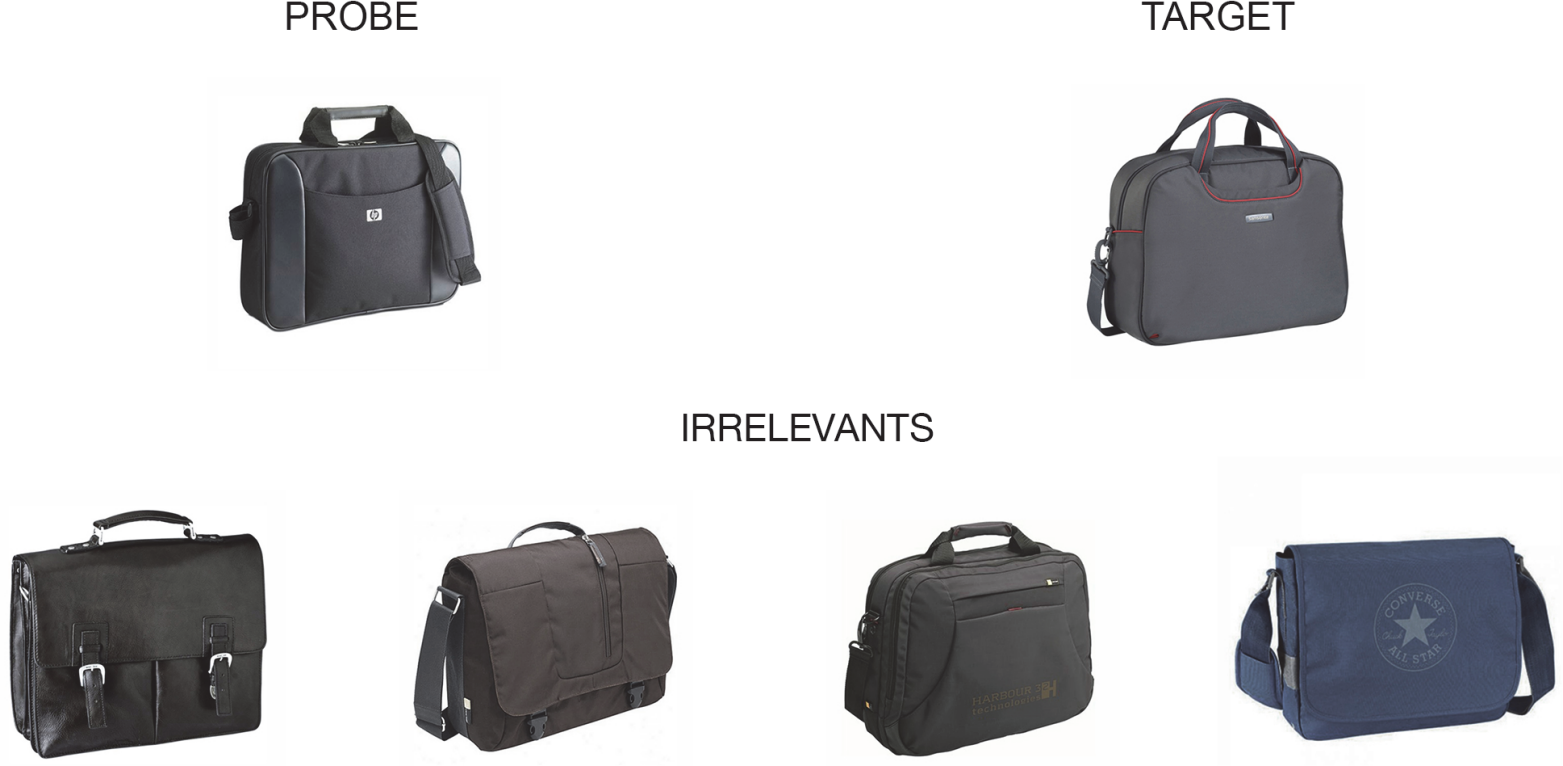
Sample items from the RT-based Concealed Information Test.

Following this filler task, each participant received written instructions to learn five items (i.e. *targets items*) that were from the same category as the probes (see **Fig. 1** for examples of CIT items from a category). Each of the five targets was presented on a computer screen for 10 seconds and the sequence was repeated three times. The instructions specified that participants had to memorize the physical characteristics of each item in order to reproduce them all later. To ensure a good retention of the target items, a verbal recall was performed.

Subsequently, each participant received another set of written instructions explaining that they were suspects of a theft and that they will undergo a behavioral test designed to assess their involvement in the crime. Also, they were informed that they will be assessed by a virtual investigator (similar to Ambach et al.,[36]).

Subjects were tested individually. They sat in front of the computer screen at a distance of approximately 50 cm in a quiet room to perform the following tasks. After completing the mock crime and the target learning phase, the participants undertook four CIT conditions designed for this study: a classical RT-CIT condition consisting in one block, and three Emotional RT-CIT conditions Em-RT-CIT, negative, positive and neutral). The order of presentation of these procedures was drawn randomly for each participant. The items used in all of the CIT procedures were pictures belonging to three categories of items: *probes* (the five critical items stolen in the mock crime), *targets* (five previously learnt items from the same category as the probes) and *irrelevants* (four items from the same category as each probe, resulting in a total of 20 items not previously encountered during the experiment).

Stimuli presentation and response acquisition were controlled by the E-Prime 2.0 software (Psychology Software Tools, Pittsburgh, PA). Subjects were instructed to respond “Yes” (“Z” key) to targets and “No” (“/” ALT key) to any other item (including probe items) by pressing the corresponding keys with their index fingers (Yes and No labels were taped to the respective keys). Answers had to be given as quickly as possible. The item remained on the screen until a response was made; if an answer was not offered within a 1200-millisecond interval, a ‘too slow’ message appeared on the screen. Three responses faster than 200 ms were removed before further analyses were conducted. The inter-stimulus interval randomly varied between 500, 800 and 1000 ms in order to discourage automatic responses or preparation effects [31]. Each of the pictures used in this study was about 15 x 15 cm.

After a short training phase, the test began. A condensed version of the instructions was displayed on the computer screen at the beginning of the testing session. Each of the four blocks included two presentations of each item, generating a total of 60 trials per block. Items were presented in a randomized order within each block.

The RT-CIT condition contained one block similar to previous versions of the test using visual stimuli [19, 25]. On each slide, the subject viewed a picture of an item and the question “Do you recognize this item?” was written below the picture, with the answers Yes and No written below on opposite sides of the screen.

**Fig 2:**
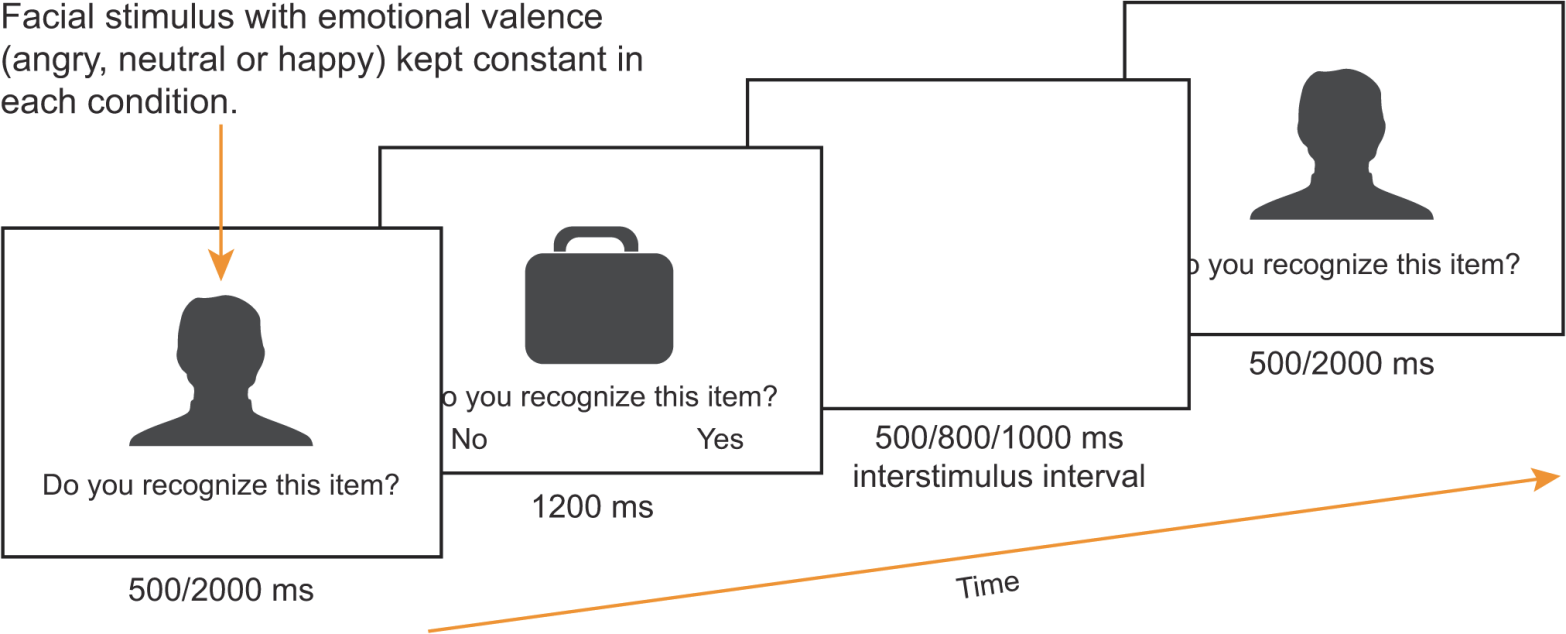
Em-RT-CIT conditions, in which the “virtual investigator” appeared before the presentation of each CIT item.

In the three Em-RT-CIT conditions, a photo of a “virtual investigator” appeared on the screen before the presentation of each item (see **Fig. 2**). On the same slide the question was shown: “Do you recognize this item?”. The slide’s duration was either 500 ms or 2000 ms (randomly) and no action was required to be performed by the participant. For this condition, an image of a middle-aged man was used from the FACES 3.3.1. database [69]. Within each block (positive, negative, or neutral), the facial expression of the investigator was happy, angry, and neutral, respectively. The selected photo for the virtual investigator (Person-ID:026), had a very high mean percentage of correct expression identification: 98% for happy, 97% for anger, and 97% for neutral [69]. Besides this screen presenting the virtual investigator, all task requirements and screen displays containing CIT items were identical to the classical RT-CIT condition.

Participants completed the four CIT conditions presented in a randomized order automatically determined by E-Prime (see S1 Appendix for a full description of the randomization procedure). After a short break, the DASS Questionaire [70] was administered.

Emotional n-back

**Fig 3:**
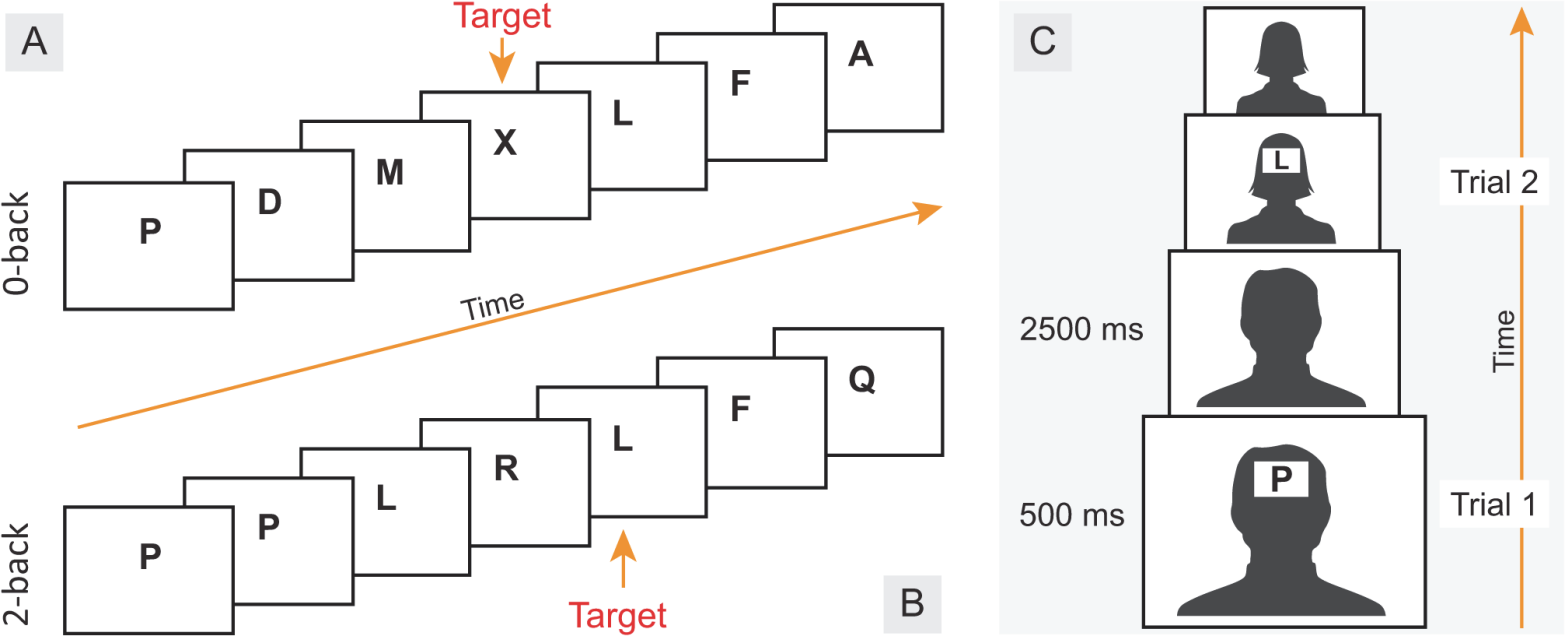
Illustration of the n-back tasks. **A.** The target in the 0-back control condition is any letter designated in the instructions (e.g., X). **B.** the target in the 2-back memory-load condition is any repeat of a letter presented two trials back (e.g., L). **C**. The EM-*n*-back task consisted of superimposing the original *n*-back task onto one of four backgrounds (no background, negative face stimuli, neutral face stimuli, and positive face stimuli.

The EM-*n*-back task consisted of superimposing the original *n*-back task onto one of four backgrounds: no background (blank screen), negative background, neutral background or, positive background (see **Fig. 3**) [61, 71]. However, following the suggestion of Casey et al., [71], who specified that in order to control for the different complexity of the pictures used as backgrounds, it would be preferable to include faces rather than natural scenes as backgrounds, we reverted to the use of facial expressions onto which the *n*-back items were superimposed. This also allowed us to better compare performance (accuracy, RT) on the EM-*n*-back blocks to the emotionally valenced blocks used in the Em-RT-CIT conditions which also involved facial expressions.

E-Prime 2.0 software (Psychology Software Tools, Pittsburgh, PA) was used for item presentation and response time recording. Participants received verbal and on-screen instructions which described that they were going to see a series of letters presented one at a time, over a background consisting of facial pictures or over a blank screen (depending on the condition). The photos depicting facial expressions were selected from the same FACES 3.3.1. database [69], but excluded the identity of the “virtual investigator” presented in the Em-RT-CIT. These included various individuals of three age groups (young, middle-age and old), male and female. The same emotional valence was maintained during each condition (i.e., angry, happy or neutral), except for the no-background condition. There were eight blocked conditions comprising two memory-load conditions (i.e., 0-back and 2-back) by four background conditions (none, negative, neutral, positive). The blocked conditions included 20 trials each, for a total of 160 trials.

For the 0-back condition, participants were asked to press a button when presented with a specific letter (e.g., X). In the 2-back condition, the target was any letter that was identical to the one presented two trials back. An example for the instructions (e.g., A-F-A) was provided on the computer screen. After a short training phase, the test began. Each trial consisted of the simultaneous presentation of a letter and a blank screen or facial stimulus (see **Fig. 3**). Letters and corresponding backgrounds appeared for 500 ms and then disappeared, leaving only the picture or blank screen visible for another 2500 ms. Trials were randomized within each block; faces stimuli were randomized within each background condition and letters were randomized across trials. The duration of the task was approximately 15 minutes.

Questionnaires

The *Behavior Rating Inventory of Executive Function—Adult version* (BRIEF-A, [72]) is a widely used instrument in clinical studies or for the assessment of individual differences in executive functioning. It is focused on evaluating the everyday behavioral manifestations of executive functioning, and consists of nine clinical scales that assess behavioral, emotional and metacognitive skills. It comprises 75 items and it usually takes 10–15 minutes to complete. The instrument offers two indexes: Behavioral regulation Index—BRI, and the Metacognitive Index—MI. The BRI is composed of four subscales: Inhibit, Shift, Emotional Control and Self Monitoring, and the MI is composed of five subscales: Initiate, Working Memory, Plan/Organize, Task Monitor and Organization of Materials. A global score, the Global Executive Composite—GEC, can be computed as a composite of the above two. The advantage of using this instrument is that it offers a detailed image of executive control in ecological, real life contexts, the contexts in which the deception is most likely to occur.

The *Depression Anxiety Stress Scales* (DASS, [70]) is widely used measure of negative affect in adults. DASS 21 is the short version of the original 42 item DASS and consists in three subscales: Depression, Anxiety and Tension/Stress, each formed of seven items taken from DASS 42. DASS is a reliable and valid measure of depression, anxiety and tension/stress in clinical and non-clinical populations, both in its original form of 42 items and in its 21 items short form [73]. For the present study’s screening purposes we considered that the short form of the instrument was more than adequate and focused on the depression and anxiety dimensions of the instrument.

The *State-Trait Anxiety Inventory* (STAI, [67]) is a 40-item self report questionnaire, using a 4-point Likert scale for each item. The scale is used to measure both *trait anxiety* (how anxious a person feels across time and situations) and *state anxiety* (how anxious a person is feeling at a particular moment) as it consists of two separate sub-scales (STAI-T and STAI-S, respectively), each with 20 items. The instrument was used in the present study in order to control for the anxiety effects (if any) in the sample.

## Results

### RT-CIT and Em-RT-CIT performance

Group effects

The differences in response latencies and accuracy between probes and irrelevants were analyzed following a similar strategy as other studies using the RT-based CIT [31–33, 19, 24]. Descriptive data for RT and accuracy according to stimulus type (probes, irrelevants and targets) and condition (RT-CIT, Neg RT-CIT, Neu RT-CIT and Pos RT-CIT) are presented in Table 1.

**Table 1:** Descriptive statistics for the mean reaction time and accuracy for the stimuli (probes, irrelevants and targets) according to the four conditions: RT-CIT, Neg RT-CIT, Neu RT-CIT, Pos RT-CIT.

	RT: Mean (SD)			Accuracy: percent correct (SD)		
	Probes	Irrelevants	Targets	Probes	Irrelevants	Targets
**RT-CIT**	671 (86)	590 (67)	687 (70)	84 (20)	97 (3)	87 (11)
**Neg RT-CIT**	678 (87)	608 (76)	716 (72)	87 (19)	97 (4)	82 (13)
**Neu RT-CIT**	707 (88)	617 (60)	714 (82)	86 (16)	96 (3)	84 (15)
**Pos RT-CIT**	717 (76)	618 (68)	720 (82)	87 (19)	96 (3)	87 (13)

We first analyzed the ***RT data*** for the correct responses, as this is considered the main output from the RT-based CIT [74]. A preliminary analysis with presentation duration of the “virtual investigator” face (500 vs. 2000 ms) revealed non-significant differences between subsequent responses to CIT items across the four conditions, so this variable was omitted from the final analysis.

A repeated measures mixed-model analysis of variance (ANOVA) was conducted, with intercept, Condition (RT-CIT, Neg RT-CIT, Neu RT-CIT, and Pos RT-CIT) and Stimulus Type (probes vs. irrelevants) and their interaction as fixed factors, and with intercept and Block Order as random factors. The main effects model revealed a significant Condition effect, F(3,44.3) = 6.42, p = .001, Stimulus Type effect, F(1,45) = 171.03, p = .001 and an interaction effect between Condition x Stimulus Type, F (3, 45) = 3.22, p = .03. The Block Order random effect was not significant (*Wald Z* = 1.18, p = .24).

*Posthoc* pairwise comparisons (with Fisher's least significant difference—LSD) indicated that subjects were significantly faster on the traditional RT-CIT than on both Neu RT-CIT and Pos RT-CIT, p < 0.02. Also, RTs were faster on the Neg RT-CIT than on the Pos RT-CIT, p < .01, yet were not significantly different from the speed of responses on the RT-CIT (p = .36) or Neu RT-CIT (p = .09). Across conditions, subjects were faster in responding to irrelevants than to probes, p < .001.

**Fig 4:**
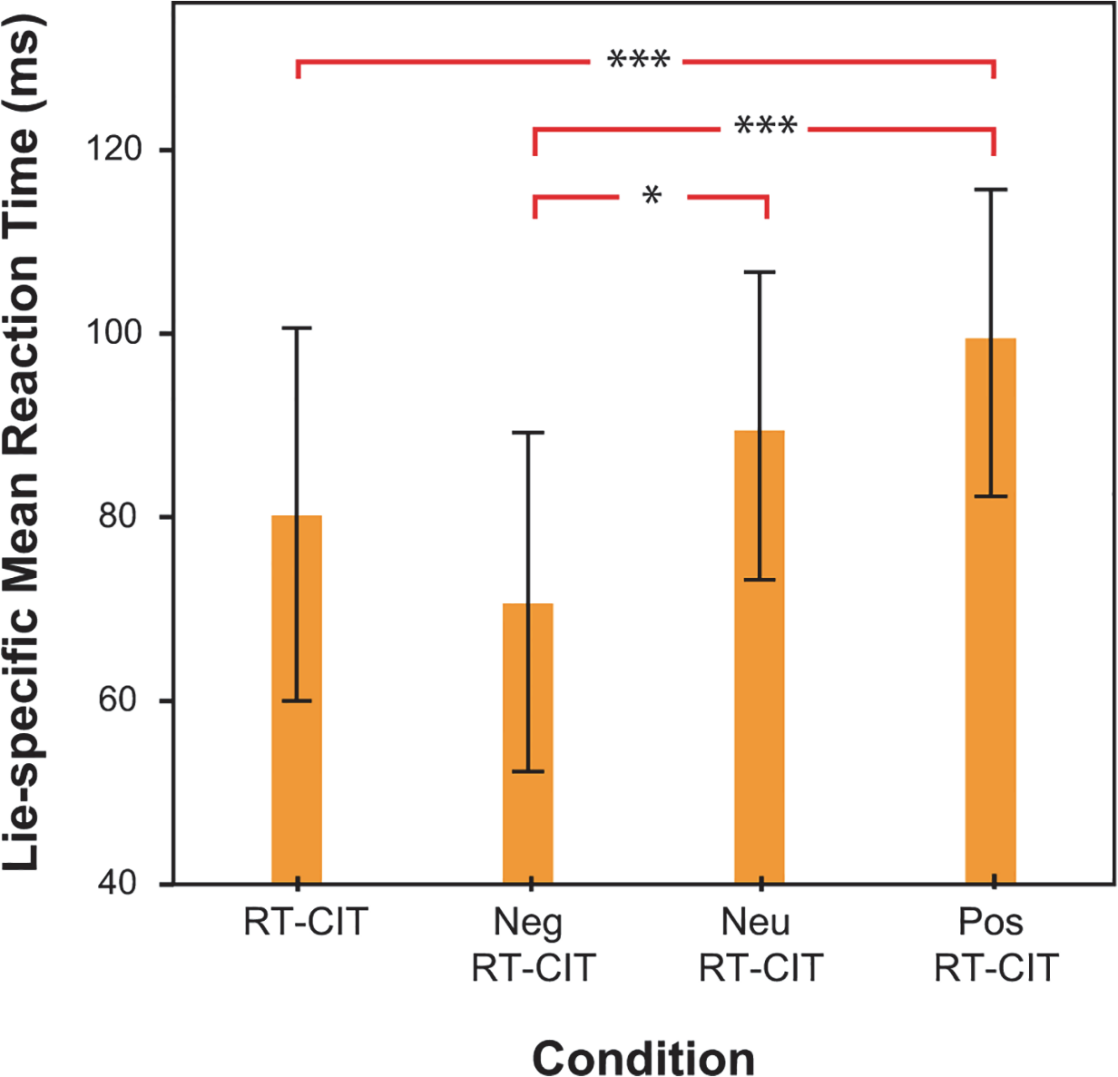
Lie-specific Mean Reaction Time for RT-CIT, Neg RT-CIT, Neu RT-CIT, Pos RT-CIT. Asterisks indicate significant post-hoc differences. Error bars indicate standard error of the mean (±2 SEM).

In order to analyze the Condition x Stimulus type interaction, again pairwise comparisons were conducted. These post-hoc analyses first confirmed that within each condition, responses to probes were significantly slower than those to irrelevants, p < .001. Next, we looked at the different speed of responses for each stimulus type (probe or irrelevant) between various conditions. When analyzing responses to *probes*, we found they were faster in the RT-CIT condition compared to the Neu RT-CIT or the Pos RT-CIT conditions, p <.05. Also, responses to probes were faster in the Neg RT-CIT condition versus the Neu RT-CIT (p < .03) or the Pos RT-CIT (p < .01). There were no significant differences in responses to probes between RT-CIT and the Neg RT-CIT. Responses to *irrelevants* were also faster in the RT-CIT condition versus the Neu RT-CIT (p < .02) or the Pos RT-CIT conditions (p < .01). There were no significant differences regarding the responses to irrelevants between Neg RT-CIT and any of the other conditions. The interaction between Condition and Stimulus type was probably driven by probe-irrelevant differences, constituting the measure of appended “lie-specific” time. Further posthoc pairwise comparisons (with Fisher's least significant difference—LSD) indicated that the difference between mean RTs for probes minus mean RTs for irrelevants was higher in the Pos RT-CIT condition than in the Neg RT-CIT, p < .01, or in the standard RT-CIT, p < .01 (see **Fig. 4**). This difference was also higher in the Neu RT-CIT compared to the Neg RT-CIT, p < .04. No significant differences were found between RT-CIT and Neu RT-CIT (p = .43), nor between RT-CIT and Neg RT-CIT (p = .38).

Another measure of detection used in the CIT is the response ***accuracy*** [31]. After we computed the percentage of correct responses according to the type of stimulus (probes, irrelevants, targets), we calculated the mean percent correct responses for probes, irrelevants and targets (see Table 1). An arcsine transformation was applied to the accuracy data in order to directly compare percentages for the probes and irrelevants [75], cf. [34]. We conducted a two-way repeated-measures ANOVA with Condition (RT-CIT vs. Neg RT-CIT, Neu RT-CIT, and Pos RT-CIT) and Stimulus type (*probes* vs. *irrelevants*) as within-subject factors. The results showed that there was a significant effect of Stimulus type, F(1, 45) = 29.84, p < .001, MSE = .08, η_p_^2^ = .39, with more errors in response to probes compared to irrelevants. There were no significant effects regarding the Condition (F(3, 135) = .61, p = .60) or the interaction between Condition and Stimulus type (F(3,135) = 1.11, p = .34).

Intraindividual bootstrap analysis

To determine the number of participants who showed a reliable probe—irrelevant difference, data from each condition were bootstrapped [76]. This procedure generates a distribution for each stimulus type within each individual, allowing for statistical testing and for computing hit rates. Basically, the bootstrapping analysis allows generating multiple different averages from the same set of stimuli [77]. After excluding incorrect behavioral responses and artifacts, a computer software draws (with replacement) a set of individual probe reaction times equal to the number of accepted probe trials in each block and also draws (with replacement) an equal number of irrelevant RTs, selected randomly from the irrelevant trials. Then, by subtracting the mean irrelevant reaction times from the mean probe reaction times, a difference score is computed. This process is repeated 500 times [33], resulting in a distribution of 500 differences scores. If the mean difference score minus 1.29 times the standard deviation is greater than zero, it can be concluded with 90% confidence that the probe reaction times are slower than the irrelevant ones.

Bootstrapping of the RT-CIT reaction times resulted in a hit rate of 71%, i.e., for 33 out of 46 participants concealed information was detectable through their slower responses on probe stimuli. For the Neg RT-CIT, a hit rate of 69% was computed, while for Neu RT-CIT, 73% of the participants displayed a reaction time for probes that sufficiently deviated from that for irrelevant stimuli to be of diagnostic value. The highest hit rate resulted for Pos RT-CIT, 78% of participants were detected in this condition.

### *n*-back and EM-*n*-back performance

**Fig 5:**
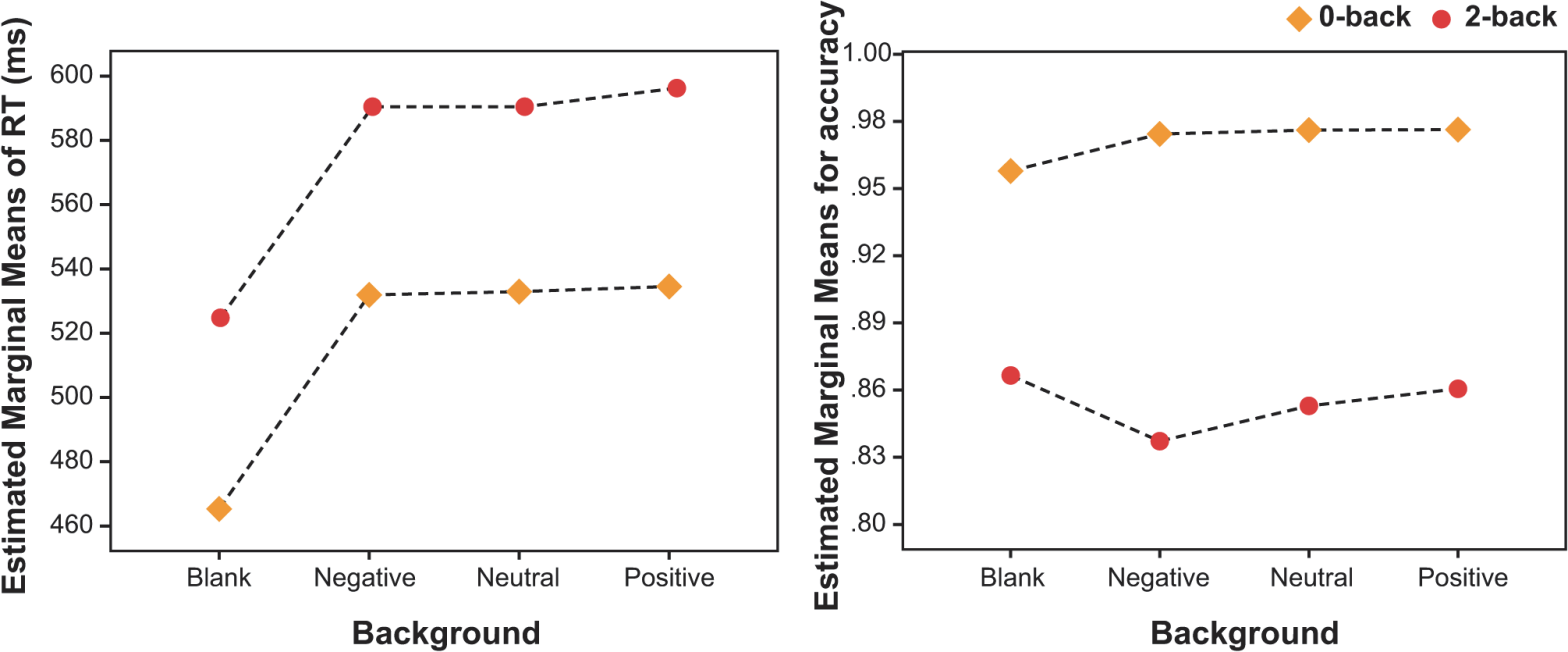
Estimated marginal means of correct-trial reaction time and of accuracy, for the EM-n-back conditions.

RT data from the *n*-back task were filtered by removing all error trials faster than 100 ms and those greater than 1000 ms [61]. The mean correct-trial RT and total accuracy score were then calculated for each task (0-back and 2-back) and conditions (no background, negative background, neutral back and positive background). Descriptive data are presented in **Fig. 5**.

A two-way repeated-measures ANOVA with Task (0-back and 2-back) and Condition (no background, negative background, neutral background and positive background) as within-subject factors was conducted for the mean RT data. The results showed that there was a significant effect of Task, F(1, 45) = 163.44, p < .001, MSE = 7889.9, η_p_^2^ = .78. Subjects were faster in responding to the 0-back task, compared to the 2-back task. There was also a significant effect of Condition, F(3, 135) = 68.4, p < .001, MSE = 1547.8, η_p_^2^ = .60. *Posthoc* pairwise comparisons (with Fisher's least significant difference—LSD) indicated that subjects were faster on both tasks on the no background condition, than on those with emotional backgrounds (p < .001). No significant difference was found between the emotional background conditions.

A two-way repeated-measures ANOVA with Task (0-back and 2-back) and Condition (no background, negative background, neutral background and positive background) for accuracy data indicated a significant effect of Task, F(1, 45) = 139.55, p < .001, MSE = .009, η_p_^2^ = .75. Subjects had better performances on 0-back than on 2-back. Again, no significant differences were found between the background conditions. For the Task x Condition interaction, there was a significant effect, F(3, 135) = 3.47, p < .02, MSE = .002, η_p_^2^ = .07. *Posthoc* pairwise comparisons (with Fisher's least significant difference—LSD) indicated that subjects had better performances in 0-back on the background conditions than on the blank condition, p < .001. However, no significant differences were found between conditions in the 2-back task.

### Role of individual differences in executive functioning and internalizing symptoms

In order to obtain a unitary individual differences measure of the appended lie-time [26] we relied on the difference between mean of responses to probe and irrelevant items [24]. Higher values of this difference indicate a longer lie-specific time, and thus a better detection efficiency of the RT-CIT. The next step was to relate the main RT-CIT outcomes (speed of responses to probes and appended lie-time) in each condition to individual differences in executive functioning, anxiety, and depression. Regarding the self-report assessment of individual differences in executive functioning measured with the BRIEF-A, there were some marginal relations with deception outcomes which failed to reach significance. Thus, these measures were not included in further statistical analysis.

In order to determine the interrelationships between proficiency in WM updating and in concealing information, Pearson correlations were computed between performance on the *n*-back task (mean RTs and accuracy scores for 0-back, 2-back) and on the RT-based CIT (mean RTs for deceptive responses to probes in CIT, and the appended lie-specific time), separately for each of their conditions (no emotion, positive, negative and neutral). Regarding the mean RTs, we found a significant positive relation, r(46) = .30, p < .05, between speed of responses to standard RT-CIT probes and speed of responses on the 0-back, no background condition. Also, mean RTs for responses on the standard 2-back (no background condition) were positively related with those on RT-CIT probes, r(46) = .30, p < .05. No other significant correlations were found between the conditions of WM tasks and speed of responses on the corresponding conditions of the CIT. In terms of accuracy, no significant relations were found between similar WM and CIT conditions. Correlations between lie-specific times on the CIT and *n*-back performance measures were also non-significant.

**Fig 6:**
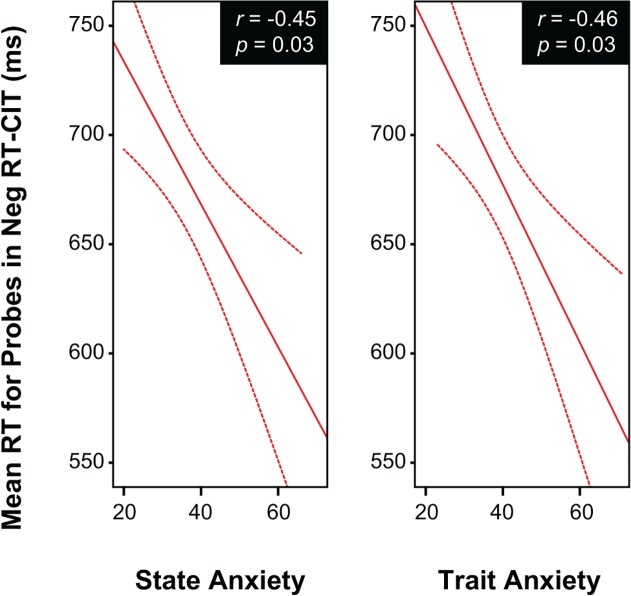
Estimated fit lines and their 95% confidence interval for the relation between RTs to probes and individual differences in state anxiety, trait anxiety.

In order to investigate relations between deception efficiency measures and individual differences in anxiety and depression we performed tests which directly assessed the hypotheses put forward (thus decreasing the Familywise error rate). Thus, adjusted multiple correlations between anxiety, depression and deception measures (RTs to probes and irrelevants) were computed (see Table 2). Regarding internalizing symptoms, responses to probes in the Neg RT-CIT was negatively related with state anxiety (StaiState), r(46) = -.45, p < .03, and also with trait anxiety (StaiTrait), r(46) = -.46, p < .03 (see also **Fig. 6**). Therefore, the association between anxiety and speed of deceptive responses seems to be circumscribed to the Neg RT-CIT and does not affect truthful responses.

**Table 2:** Adjusted multiple correlations between anxiety, depression and deception measures: RTs to probes and irrelevants.

	Pearson r (adjusted *p)*		
	StaiState	StaiTrait	DASSDepression
**Probes**			
RT-CIT	-0.03 (1)	0.09 (1)	0.18 (1)
Neg RT-CIT	-0.45 (0.03)	-0.46 (0.03)	-0.27 (1)
Neu RT-CIT	-0.18 (1)	-0.12 (1)	0.09 (1)
Pos RT-CIT	-0.07 (1)	-0.24 (1)	-0.06 (1)
**Irrelevants**			
RT-CIT	0.07 (1)	0.12 (1)	-0.05 (1)
Neg RT-CIT	-0.17 (1)	-0.20 (1)	-0.22 (1)
Neu RT-CIT	-0.17 (1)	-0.05 (1)	-0.01(1)
Pos RT-CIT	-0.00 (1)	-0.11 (1)	-0.17 (1)

Note: The adjustment of the *p-value* was computed with Bonferroni correction. If the adjusted *p*-value exceeded 1, it was set to 1. State anxiety (StaiState), Trait anxiety (StaiTrait), DASS Depression subscale (DASSDepression).

The negative relationship with the probes in the Neg RT-CIT did not reach significance in the case of the DASS Depression subscale r(46) = .18, p = 1 No significant relations were found between the other conditions of RT-CIT and anxiety or depression measures.

Considering this pattern of association between higher levels of state anxiety (the strongest among the relations with internalizing symptoms) and speeded responses to probes in the negative condition, we re-conducted the initial repeated measures ANOVA with Condition (RT-CIT vs. Neg RT-CIT, Neu RT-CIT, and Pos RT-CIT) and Stimulus type (*probes* vs. *irrelevants*), this time introducing the state anxiety score as a covariate. All previous effects (main effects of Condition and Stimulus Type) remained valid, with the exception of the Condition X Stimulus type interaction, which was no longer significant, F(3,129) = .83, p = .48 when entering state anxiety as a covariate. From the previous correlation analysis (see Table 1), we noted the selective association between state anxiety and speed of responses to probes (r = -.46) (and not to irrelevants, r = -0.17) in this negative (and not in any other) condition of the RT-CIT. However, probably due to the reduced statistical power of the analyses, this correlation was only qualified by a marginally significant interaction between Stimulus type and the state anxiety score, F(1, 43) = 3.21, p = 0.08, or between Condition and the state anxiety score, F(3, 129) = 2.38, p = .07.

We also checked whether the same relationship between anxiety and depression scores would hold true in the case of the *n*-back task. We only found some positive relations between the state anxiety score and latency of responses in the positive (r = .27), negative (r = .26) and neutral (r = .33) conditions of the EM-*n*-back, which did not remain significant after the Bonferroni correction.

## Discussion

The current study is based on the view that deception is an embodied and embedded process, integrating cognitive (e.g. executive functions), socio-emotional and contextual factors. We explored whether introducing a social stimulus that simulates a virtual investigator would facilitate the detection of concealed information, for the first time in a mock-crime based RT-CIT. Also, we studied how the emotional expressions of this virtual investigator would influence responses to crime-relevant items, compared to irrelevant items. Besides its influence on the RT-CIT, we investigated the impact that such emotional distraction have on a task measuring the continuous updating of WM representations, a skill that has been documented to be essential for deceptive behavior. Finally, extending the emerging literature on the impact of individual differences in cognitive and affective or personality variables on deception [18–20, 78, 79], we wanted to explore the relationship between self-report measures of executive (dys)functions, internalizing problems (anxiety and depression) and the efficiency of deceptive behavior. Several preliminary findings have resulted, which support the need to further explore the relevance of introducing emotional valence in the RT-based CIT in order to enhance its ecological validity, and not ultimately, its detection power.

First of all, we needed to verify whether the classical RT-based CIT with visual stimuli developed for the purpose of this study, and especially the modified versions containing the emotional stimuli would be able to elicit the documented “concealed/guilty knowledge effect” [31]. More specific, responses to critical visual stimuli derived from a mock crime in the RT-based CIT have been documented to elicit longer RTs and a higher error rate, compared to responses to newly encountered visual stimuli from the same category [19, 25, 32]. In the present scenario, across conditions, subjects presented longer RTs and lower accuracy on the probes, compared to the irrelevants. Individual detection rates based on bootstrapping methods showed an adequate overall RT-CIT hit rate for all of the conditions, supporting the potential of the standard RT-CIT and of the Em RT-CIT conditions to detect concealed information.

Next, we set out to explore potential differences in the discriminatory power of the four CIT conditions: one classical, one containing a social stimulus (“virtual investigator”) with a neutral facial expression [36], and two new conditions presenting the facial display of a social stimulus with emotional expressions (friendly/happy or hostile/angry). One of the main advantages of using the CIT as a paradigm to elicit and detect deception resides in its simplified nature, which eliminates some of the most complex executive processes involved in deceptive scenarios [80], such as decision to lie (already imposed by the initial instruction) or construction of deceptive responses (replaced here by simple Yes/No answers). Even in this simplified context, the “lie-specific” RT represents a sum of multiple cognitive processes such as basic attention selection and orienting mechanisms towards salient/recognized stimuli [81], inhibitory/conflict resolution mechanisms involved in selecting the deceptive response over the prepotent truthful one [25], permanent information updating [22] and switching between truthful and deceptive responses [25]. So any potential social/emotional influence from the stimulus depicting a “virtual investigator” needs to be viewed through the lens of the growing literature dealing with socio-emotional influences on attention and cognitive control [82–84].

With these considerations in mind, a preliminary observation derived from the present study is that RTs to probes and irrelevants were faster in the classical RT-CIT compared to the emotional versions (except for the non-significant comparison with the Neg RT-CIT). However, this difference between conditions was not translated in the accuracy of responses across various conditions. This suggests that although RT-CIT performance per se was not impaired by the social/emotional distractors, there was interference between their presence and the speed of participants’ responses. Thus, in the presence of neutral or positive emotional displays from a virtual investigator, participants took significantly longer to respond in the CIT, compared to a condition lacking such an examiner. A straightforward interpretation of this result is that the introduction of the virtual investigator’s face could simply act as a distractor which promotes attentional lapses, disrupts goal maintenance [23] and thus results in an overall decrease in response speed. However, since this effect was not homogenously distributed across the three conditions, and did not equally affect responses to probes and irrelevants in each condition, a more nuanced view is possible, taking into account the role played by emotional valence, and possibly arousal/motivational relevance assigned to this stimulus.

The fact that presenting an emotionally *neutral* facial stimulus representing a virtual investigator impaired CIT speed could have another explanation, besides the simple goal maintenance disruption described above. The simple presence of a virtual investigator continuously reinforces the deception detection scenario and thus assigns particular significance to the deception task, making the subjects more cautious in their responses. However, this effect did not selectively impair responses to probes. Also, in our case, the lie-specific time was *not* significantly longer in the condition involving a social stimulus displaying neutral affect compared to the classical RT-based CIT. It has been shown that a greater motivation to remain undetected is related to enhanced differential responding to probes versus irrelevant items in the CIT [29]. In the Ambach et al. [36] study investigating physiological and behavioral correlates of deception in a CIT context, the introduction of a face and voice assigned to a “virtual investigator” resulted in an enhancement of differential responding (cardiac, pulmonary, vascular, yet not electrodermal) to probes compared to irrelevant items. Considering the essential differences between the physiological CIT and the RT-based CIT (different stimulus and response timing, blocked vs. randomized presentation of stimuli), and the fact that in the Ambach et al. [36] study the facial stimulus was accompanied by a voice, which was not the case in our design, the results of the two studies are not directly comparable. Thus, the mere presence of a neutral social stimulus simulating a virtual investigator interferes with the RT-CIT by disrupting overall goal maintenance or by making subjects adopt a more cautions response style, yet this manipulation does not generate differential responding to critical versus irrelevant items, and thus does not significantly improve RT-CIT detection efficiency.

The next step was to analyze the two conditions containing emotional facial expressions. Emotion and cognition are intrinsically tied in realistic deception scenarios, and their interplay has not yet been systematically evaluated in controlled experimental settings [85]. Considering that emotion distraction has been shown to act as a “double-edged sword” that can either enhance or hinder cognitive performance [86], especially when motivational relevance can be attributed to it [57], no a-priori hypotheses have been advanced with regard to the impact of a specific emotion on CIT performance.

Surprisingly, examination of the group data revealed that there were no differences in overall speed of responses when comparing the RT-CIT to the *Neg RT-CIT*. Compared to the other conditions of the CIT containing the virtual investigator, the overall reaction time in the Neg RT-CIT was the only one which did not increase, even though a virtual investigator’s face was displayed in between CIT trials. The lie-specific time in this particular condition containing negative emotional distractors (angry/hostile virtual investigator) was also not distinguishable from that of the RT-CIT, yet significantly differed from the conditions containing a neutral or a positive facial expression. One possible hint explaining these effects is that the RT for deceptive responses to *probes*, and not to irrelevants, was significantly faster in the Neg RT-CIT than in the Neu RT-CIT, or the Pos RT-CIT. Thus, participants were faster in deceptive responses in the presence of a negative emotional display of the virtual investigator, compared to the condition which displayed a neutral/positive facial stimulus. What could be the reason behind this selective speeding up of deceptive, but not truthful responses in the negative condition? A first difference takes into account the different *relevance* that emotional distraction plays with regards to the two types of stimuli. While emotional stimuli are in principle task-irrelevant across all conditions (they are not related in any direct way to the CIT task), angry/hostile displays could become *relevant* threats especially in the case of deceptive, and not of truthful responses. This effect is related to the enhanced motivation to avoid potential negative consequences associated with deception detection in the presence of an angry/hostile examiner. Second, a significant difference between truthful and deceptive responses in the RT-CIT is that the latter impose a higher degree of *effortful control* [23], which could be selectively facilitated by the presence of negative emotional information. The interaction between these two types of explanations is particularly relevant, since it has been conjectured that “it may thus be that relevance in general that speeds up conflict processing, rather than emotion specifically” [83]. A final explanation could be related to interactions between *arousal*, emotional valence and attention selection/memory/cognitive control mechanisms. Negative stimuli are usually considered more arousing [87]. It has been shown that arousal can stimulate the memory consolidation of relevant information by increasing the selectivity of attention and memory for the respective arousing experiences [82]. In the presence of negative arousing information in particular, perceptual (yet not semantic) processing of the relevant stimuli has been found to be enhanced [88], which might have facilitated speed of responses on the CIT.

Finally, overall responses to the *Pos RT-CIT* condition were significantly lengthier than those in the classic RT-CIT condition. However, there were no significant differences regarding group data between Pos RT-CIT and the Neu RT-CIT condition. Individual bootstrap analysis also indicated that the highest hit rate resulted for Pos RT-CIT, 78% of participants being detected in this condition. Displaying a positive facial stimulus of the investigator increased both the time needed to respond to probes and to irrelevants, but much more so for probes, resulting in the largest difference between the two types of stimuli (98 ms).

Why would positive information impair speed of responses in this condition, and have a more pronounced effect on the probes, in an antagonistic way compared to previously discussed results in the negative RT-CIT? Again, we turn to the relevant literature on emotion-motivation-cognitive control interactions. Positive affect has been shown to result in a broadening of the scope of attentional filters, and most importantly in a type of “relaxation” of selective attention mechanisms which interfere with the ability to process conflicting information [89–91]. This broadening/non-focusing effect has been specifically emphasized in contexts of low approach motivation [57] such as the one encountered in the context of the Pos RT-CIT, in which the presence of the examiner, although friendly, could still be hardly perceived as a reward. An integrative explanation which would attempt to explain the antagonistic effects of negative versus positive facial expressions relates to the fact that, as shown above, while negative information enhances focused, perceptual processing, thereby facilitating the detection and recognition of the probes, positive emotion has been related to a broadening and relaxation of attentional networks, impairing the attention selection and classification process required by the CIT. Finally, a more speculative explanation dwells on the potential relevance which could be assigned by subjects to this emotional expression (and would explain the similarity of its effects with the neutral condition), resulting in a conflict between the threatening purpose of the virtual investigator to detect deception and his friendly or neutral facial expression. Experiencing this conflict between positive facial display and aversive motivation could create cognitive load, which might have resulted in an increase in their overall RTs, but especially in those trials for which the discrepancy between intention-display is the highest (i.e. deceptive trials). Previous studies have shown that experiencing incongruence between emotion expression and inferred emotional state or motivation (e.g. from verbal expressions, tone of voice, context) requires additional cognitive resources, translated in significantly prolonged RTs [92], which interfere with the processes of conflict resolution [93] similar to the ones required by CIT probes. A final speculative proposal is that the presence of a positive (or neutral) facial expression from an examiner might actually increase the feelings of guilt or shame that participants normally feel while deceiving in the CIT [46, 94]. Seen as an interpersonal construct [95], guilt has been proven to be amplified (at least in developmental samples) in a warm and affectionate environment, rather than in punitive and rejective ones [96]. This means that guilt might not be related to an expectation of punishment, as would be induced by the angry/hostile investigators’ face, but rather be amplified by the incongruence with his positive/neutral emotional expression. This speculative line of reasoning needs at least reports of subjective feelings of guilt and can be pursued by future studies investigating this interesting and somehow paradoxical effect that the investigator’s facial expression had on the guilty participants’ deceptive responses.

Next, we set out to compare the effect that emotional distraction would have on a standard memory updating task such as the *n*-back. Similar to the four RT-CIT conditions, we used three versions of the task in which the memory task was superimposed onto a background (facial expression), in contrast to the no-background condition [61]. A relevant finding was that again, the introduction of task-irrelevant social-emotional distraction affected RTs on the memory task, but it did not impair performance accuracy in the critical 2-back task, similar to previous results obtained with the EM-*n*-back task in typical and clinical populations [61, 97, 98]. Interestingly, there was an accuracy advantage in the 0-back task on the conditions containing emotional distractors compared to the standard version. This task simply required letter detection and displayed clear ceiling effects, with a mean accuracy ranging between 95% and 97% in all of the four conditions. The introduction of emotional backgrounds might have maintained participants focus throughout this facile task. In the memory-demanding 2-back task, just like in the RT-CIT, participants could perform equally well in the conditions containing or lacking social-emotional distracters, yet the impact of the distractors was again visible at the level of response speed. The non-specific elongation of RTs in the conditions containing faces could suggest that when simultaneously displayed with a target and a background (in contrast with the RT-CIT in which faces were presented before trials), the increasing complexity of the stimulus required more resources to be processed (a common finding in all the above mentioned papers using the EM-*n*-back). However, unlike the RT-CIT, where we saw a modulation of the effect in relation to affective valence, here the effect of emotion did not discriminate across the three conditions. It is plausible that the emotional/motivational significance assigned to the examiner might have amplified the significance of his emotional expressions in the RT-CIT—however, future studies need to explicitly test this hypothesis via self-report or psychophysiological indicators of mood induction. However, the fact that multiple facial identities were introduced in the *n*-back task, and a single facial identity in the RT-CIT does not allow us to directly contrast the results from the two tasks.

To further determine the interrelationships between WM updating and the CIT, we correlated performances on the *n*-back task (mean RTs and accuracy scores for 0-back, 2-back) with the ones on the RT-based CIT (mean RTs for deceptive responses to probes in CIT, and lie-specific time), separately for each of their conditions (no emotion, positive, negative and neutral). We found a significant relation between responses to the standard RT-CIT probes and speed of responses on the 0-back and 2-back, but only in the no background conditions. This suggests that in the absence of distracting information, similar updating skills might determine both speed of responses to critical items in the CIT and the correct identification of targets in the *n*-back. A previous study by Ambach and collaborators [21] provided evidence for the direct interference between the CIT and the *n*-back task, also suggesting a common reliance on memory updating mechanisms. An alternative, simpler possibility is that more basic mechanisms, such as individual differences in speed of processing, might underlie such interrelationships. However, no significant correlations were found between the distinct conditions of the WM task and those of the RT-CIT containing distracters, which suggests that facial/emotional distraction affected participants’ responses in distinct ways across the two tasks. Thus, it can be speculated that the motivational relevance of the virtual investigator might be responsible for the effects of his various emotional displays in the RT-CIT, although it did not seem to differentially affect performance on the *n*-back conditions, besides the common overall elongation of RTs noted on both tasks.

Another aim of the current study was to explore the modulating effect played by individual differences in executive functions and trait-like predispositions to experience symptoms of depression, anxiety, as well as in the effects of state anxiety during the RT-CIT. No significant relations were found between self-reported executive dysfunctions and deceptive responses. It is possible that the use of a non-clinical sample might not have allowed for substantial variation within scores measuring executive deficits [99]. Alternatively, it is possible that the subtle variations in executive functioning consequential for deceptive behavior are not readily captured by a self-report measure, which in other studies was at best moderately related to performance-based measures of attentional/cognitive control performance [100, 101].

Regarding the role of individual differences in state/trait anxiety, an interesting finding was their direct association with increased speed of responses to probes, and thus their negative association with lie-specific time in the negative RT-based CIT condition. This is somewhat inconsistent with studies investigating the interaction between anxiety and attentional/cognitive control, which suggest that for high-anxious subjects, attention is readily “captured” by emotional stimuli (especially by potentially threatening ones), which would lead to higher interference with the primary task, reflected in an elongation of RTs and with other indicators of reduced efficiency [62]. Interestingly, this expected tendency of high-anxious subjects (but not of those with high depressive symptoms) to present slower RTs to targets in the presence of emotional distracters was confirmed (although it did not reach statistical significance) in the EM-*n*-back task. What was different in the Em-RT-CIT which made high-anxious subjects provide faster, yet not less accurate responses selectively to probes in the negative condition of the CIT? High state anxiety, when in a typical range, can enhance participants’ motivation to perform well and their alertness toward the task, increasing their ability to focus and to provide faster responses, as was visible in our task [102]. Since the interaction between stimulus type and condition became non-significant when anxiety was introduced as a covariate, and considering the abovementioned association between faster responses to probes and anxiety, one can speculate that the selective “speeding up” of responses to critical items in the negative condition might be mediated by anxiety-related effects (although the anxiety X condition interaction failed to reach statistical significance).

Before discussing the theoretical and applied relevance of these findings, several *limitations* warrant discussion. First, using a blocked design for the emotional versions of the RT-CIT and *n*-back instead of using a trial by trial alternation design might have triggered a decrease in response to the social stimulus after repeated presentations (habituation). Indeed, the use of only one facial identity does not allow for the results to be extrapolated to the impact of facial emotional expressions in general on the RT-CIT. However, it was estimated that the choice of a single facial identity has advantages in terms of its potential analogy (which was presented as such to the participants within the Instructions, as in the Ambach et al. study [36]) with a situation in which a real investigator confronts the suspect with evidence from the crime. In this context, we were mainly interested in the impact of the emotional expression displayed by this constant virtual investigator, rather than in the more general effect of facial emotional expressions. To ensure that the present findings are not confined to the particular face stimulus we used, a further experiment using one or several different facial identities would clarify the reliability of the effect and would permit the generalization of our results.

Another option which might have reduced habituation effects besides the use of more facial identities, would have been to replace the blocked with a randomized design in terms of emotional valence. It has been shown that the immediate emotional context created by a previous trial can have a greater effect than the emotional content itself [102, 103]. If we had chosen to alternate the social stimuli within a block, the emotional valence effect on the task outcomes would be difficult, if not impossible, to isolate. The emotional valence effect from a trial could persist across the following trials, which might lead to their contamination due to the difficulty in disengaging from the previous encountered stimuli. In addition, other studies have shown that motivational/emotional effects on attention orienting and on cognitive control (especially conflict resolution) processes are usually subjected to temporal dynamics [104, 105]. Thus, while the effect of the social stimulus might decrease with habituation [106], the potential mood induction effects on attention orienting and on memory recognition remain viable throughout the task.

Second, only guilty participants were included in this study. Despite the fact that the performance of an innocent group distribution can be statistically estimated [107, 24], it is recommended to include an authentic group, rather than a simulated one. In order to disentangle the presumed motivational effects of introducing a virtual investigator with various emotional expressions, from the mood-inducing effects of facial emotional expressions, a future design needs to include such an innocent group. We partially attempted this by comparing the effects of emotional valence in the RT-CIT with the effect of emotional facial distracters in the *n*-back task—a comparison which indicated non valence-specific effects in the latter.

Third, self-report behavioral inventories, such as the BRIEF [68], might not be the optimal way of testing the executive functioning. The level of agreement between the BRIEF and well-established executive tasks is at best modest [108]. However, these inventories are often a useful adjunct to cognitive assessments as they enable behavioral and qualitative information to be collected and interpreted in a standardized format [109]. Importantly, the limited sample size (although common in RT-CIT studies which use a within-subjects design), and also the uneven distribution of the sample by gender can affect the generalization of the data from the present investigation, making its results preliminary and in need for further replication in independent samples. A final concern is regarding the unbalanced nature of the order in which the conditions were administered across the sample—however, our analyses have revealed a non-significant block order effect when treated as a random factor. Future studies should ensure that a balanced order of block administration is used in order to limit the impact of this variable.

### Conclusions & implications

While the flows of social, emotional and cognitive information are naturally intertwined in deceptive scenarios, most of the literature on deception detection has selectively focused on the impact of enhancing cognitive load on deception. The present study attempted to simulate interference from social and emotional stimuli, and to assess its impact in a controlled, laboratory-based concealed information scenario. Despite the artificial elements induced by explicitly instructing participants to commit a mock crime and to lie about it, the RT-based CIT has proven its efficiency in identifying the “memory traces” of concealed information and has the potential to represent a cost-effective method for real-life deception detection settings [74]. It is important, therefore, to introduce social and emotional information in this task and to assess to what degree this manipulation impairs/facilitates the process of deception detection. There were several intriguing findings when comparing the standard RT-CIT to the conditions containing emotionally-relevant stimuli described as expressions of a “virtual investigator” [36]. First, deceptive performance (accuracy) was similar across all conditions, and only the RT measure was sensitive to the effects of emotional valence. Second, in the presence of a social stimulus with a neutral emotional valence, although participants took longer to respond, this did not differentially affect responses to probes and to irrelevants, and thus it did not prolong the lie-specific time. This essential measure which constitutes the concealed information effect was significantly longer in the condition containing a happy/friendly examiner. In contrast with this tendency, in the condition containing an angry/hostile examiner, subjects were actually faster in responding to probes compared to all conditions containing facial stimuli, this leading to a decreased detection efficiency. While the attempted explanations are speculative and so far limited by the use of a unique facial identity (therefore not readily transferable to other facial stimuli), a replication of this finding suggesting antagonistic effects of the emotional display of a virtual investigator (positive vs. negative) would have substantial implications for interviewing techniques.

Interestingly, this contrasting effect of positive versus negative distracting information was not noted when measuring cognitive performance on a task requiring memory updating, a process proven to be relevant for the production of deceptive behavior. A simple elongation of response times (and the same absence of effects on response accuracy) was visible across the conditions of the EM-*n*-back task, compared to the standard version. This could imply that specific motivational factors assigning relevance to the emotional distractor could have differentiated between the impact of positive versus negative emotional information in the Em-RT-CIT.

To conclude, emotional valence has been shown to differentially affect speed of responses to critical items in a mock-crime derived RT-based CIT, with positive facial expressions from a virtual examiner enhancing detection efficiency, compared to a version containing negative facial expressions. The study also raises the possibility that individual differences in state/trait anxiety experienced by deceptive participants could play a motivational role, further interfering with the process of deception detection and selectively affective responses to critical items, especially in the presence of a hostile facial expression. Besides the need to replicate these effects using other facial identities, we also need to verify if these emotion-related effects generalize to the context of other established paradigms for the detection of concealed/deceptive behavior, such as more traditional versions of the CIT based on physiological measures, the Sheffield lie test [14, 110] or recent applications of the Implicit Association Test to deception detection [111]. If replicated, the findings emphasize the need to investigate motivational and emotional factors when transferring deception detection techniques from the laboratory to real-life settings [112], which usually involve affective-laden reactions circumscribed by contextual and dispositional factors such as the ones targeted by the present investigation.

## Supporting Information

S1 Appendix Description of the randomization procedure. (DOC)
