# Supplementary material for: The “Good Cop, Bad Cop” Effect in the RT-Based Concealed Information Test: Exploring the Effect of Emotional Expressions Displayed by a Virtual Investigator
Source: PLoS One. 2015 Feb 20;10(2):e0116087. doi: 10.1371/journal.pone.0116087 (PMC4336287; doi:10.1371/journal.pone.0116087)
Supplement: S1 Appendix — (DOC) [file pone.0116087.s001.doc]

**APPENDIX S1**

**Table 3.** Description of the randomization procedure. The table shows the number of occurrences of the conditions according to each experimental block.

|  | **1st Block** | **2nd Block** | **3rd Block** | **4th Block** |
| --- | --- | --- | --- | --- |
| **RT-CIT** | 11 | 7 | 14 | 14 |
| **Neg RT-CIT** | 10 | 14 | 12 | 10 |
| **Neu RT-CIT** | 12 | 17 | 8 | 9 |
| **Pos RT-CIT** | 13 | 8 | 12 | 13 |
